# Induction of Multi-Functional T Cells in a Phase I Clinical Trial of Dendritic Cell Immunotherapy in Hepatitis C Virus Infected Individuals

**DOI:** 10.1371/journal.pone.0039368

**Published:** 2012-08-14

**Authors:** Shuo Li, Stuart Roberts, Magdalena Plebanski, Maelenn Gouillou, Tim Spelman, Philippe Latour, David Jackson, Lorena Brown, Rosemary L. Sparrow, H. Miles Prince, Derek Hart, Bruce E. Loveland, Eric J. Gowans

**Affiliations:** 1 Burnet Institute, Melbourne, Victoria, Australia; 2 Department of Immunology, Monash University, Melbourne, Victoria, Australia; 3 Alfred Hospital, Melbourne, Victoria, Australia; 4 School of Public Health and Preventive Medicine, Monash University, Melbourne, Australia; 5 Department of Microbiology and Immunology, The University of Melbourne, Parkville, Victoria, Australia; 6 Australian Red Cross Blood Service, Melbourne, Victoria, Australia; 7 Centre for Blood Cell Therapies, Peter MacCallum Cancer Centre, East Melbourne and University of Melbourne, Victoria, Australia; 8 The University of Sydney, Sydney, New South Wales, Australia; 9 Department of Microbiology, Monash University, Melbourne, Victoria, Australia; 10 Discipline of Surgery, The University of Adelaide, Adelaide, South Australia, Australia; University of Montreal, Canada

## Abstract

We have previously reported a world-first phase I clinical trial to treat HCV patients using monocyte-derived dendritic cells (Mo-DC) loaded with HCV-specific lipopeptides. While the brief treatment proved to be safe, it failed to reduce the viral load and induced only transient cell-mediated immune responses, measured by IFNγ ELIspot. Here we reanalysed the PBMC samples from this trial to further elucidate the immunological events associated with the Mo-DC therapy. We found that HCV-specific single- and multi-cytokine secreting T cells were induced by the Mo-DC immunotherapy in some patients, although at irregular intervals and not consistently directed to the same HCV antigen. Despite the vaccination, the responses were generally poor in quality and comprised of primarily single-cytokine secreting cells. The frequency of FOXP3^+^ regulatory T cells (Treg) fluctuated following DC infusion and eventually dropped to below baseline by week 12, an interesting trend suggesting that the vaccination may have resulted in a more subtle outcome than was initially apparent. Our data suggested that Mo-DC therapy induced complex immune responses *in vivo* that may or may not lead to clinical benefit.

## Introduction

Hepatitis C virus (HCV) persistently infects ∼3% of the world's population, leading to cirrhosis, cancer and liver failure. Despite the burden of this disease, no vaccine is available and treatment options still remain limited, particularly in those who fail to respond to the current standard of care. We have previously completed a Phase I clinical trial to treat HCV patients using dendritic cell therapy [1]. In this trial, Mo-DC from HLA-A2 positive, HCV genotype 1 infected patients, who had previously failed interferon-based therapy, were generated *ex vivo* [1]. The Mo-DC were pulsed with six lipopeptides comprised of HLA-A2-restricted HCV-specific cytotoxic T lymphocyte (CTL) epitopes, each linked to a universal Th epitope, and to the TLR2 agonist Pam_2_Cys [2]. The cells were exposed to prostaglandin E2 then infused into the patient by the intravenous and/or intradermal routes. Patients received 1, 2 or 3 infusions at 2 weekly intervals. While all patients tolerated the injections well, there were no dose- or time-dependent reductions in the HCV viral load following the DC infusions. Analysis of the T cell response, determined by the secretion of IFNγ and measured by ELIspot, showed that only relatively weak anti-HCV immunity was induced, as only transient and sporadic cell mediated responses were detected that appeared to have no apparent correlation with the dose or the timing of the DC infusions.

To our knowledge this is the first and only trial of DC therapy in HCV patients. Induction of HCV-specific T cells was measured as IFNγ-producing cells by ELIspot assay as this was considered to be the gold standard for immunological assessment in the clinical setting. However, the induction of multi-functional effector T cells, which produce multiple cytokines simultaneously, are believed to be critical for immune defence against pathogens [3] and current opinion is that this should be borne in mind for vaccine development [4]. Consequently, to assess the potential of Mo-DC therapy it is critical to determine if multi-functional effector cells were induced.

The aim of this study was to re-examine PBMC from patients in this trial for evidence of multi-functional effector T cells and/or any change in the frequency of regulatory T cells that might help explain the low levels of induced immune responses.

## Materials and Methods

### Patients and cells

A total of 6 patients were treated in the trial as described previously [1]. PBMC analysed here were sampled before and after Mo-DC infusion and cryo-preserved. Cryo-preserved PBMC from 5 healthy donors (Australian Red Cross Blood Service) served as controls. The study was approved by the Alfred Hospital Human Research Ethics Committee and the Victorian Department of Human Services Human Research Ethics Committee. Written informed consent was obtained from each patient and volunteer.

### Antigens

The HCV peptide array for genotype 1a, which contains 18-mer peptides overlapping by 11aa, was provided by BEI Resources, ATCC. Peptide pools (pp) corresponding to the HCV core, E1/E2, P7/NS2, NS3, NS4A/4B, NS5A and NS5B proteins were prepared as working stocks of 100 µg/ml in DMSO/RPMI and the final concentration in culture was 2 µg/ml. The 6 HCV CTL epitopes contained in the cellular vaccine (HCV core 35 YLLPRRGPRL, core 132 DLMGYIPLV, core 177 FLLALLSCLTV, NS3 1406 KLVALGINAV, NS4B 1807 LLFNILGGWV and NS4B 1851 ILAGYGAGV) were pooled and the final concentration for each in the culture, and for P25, the universal Th epitope, was 2 µg/ml. Positive controls comprised 2 µg/ml CEF peptide pool (containing major CTL epitopes of human cytomegalovirus, Epstein–Barr virus and influenza A virus [5]).

### Antibodies

FOXP3 antibody, its isotype control antibody and staining buffers were purchased from eBiosciences (San Diego, US), and all the other antibodies and isotype controls were purchased from BD Biosciences (New Jersey, US)

### Intracellular staining

ICS was performed essentially as described previously [6], [7]. In brief, cryopreserved PBMC were thawed into warm RPMI-1640 supplemented with 10 U/ml DNase (Sigma-Aldrich, Castle Hill, NSW, Australia), and washed 2 times in this medium. The cells were then resuspended (5×10^6^ PBMC/mL) in complete medium containing RPMI-1640, 2 mM L-glutamine, 100 IU/mL penicillin-streptomycin (Invitrogen, Mount Waverley, VIC, Australia) and 10% FBS (Thermo Fisher Scientific, Scoresby, Victoria, Australia) and rested overnight (∼16 h) at 37°C with 5% CO_2_.

The cells were washed and resuspended in a 96-well U-bottom plate in complete medium. Test or control antigens were added to a final concentration of 2 µg/mL and the cultures incubated for 6 hours, with BrefeldinA and Monensin (eBiosciences) supplementation for the final 5 hours. After stimulation, the cells were first stained with ViViD (Invitrogen) to discriminate dead cells, followed by surface staining for CD3 and CD4 and then intracellular staining of IFN-γ, TNFα and IL-2. A small number of cells were used immediately after resting (without antigen stimulation) for detection of FOXP3 as we described previously [8]. Flow cytometry was performed using BD SLRII, and data were analysed using FlowJo online software. Data are expressed as the percentage of cytokine producing cells within the gated viable T cells, background-subtracted using the no antigen control of the same PBMC sample. When the mean fluorescent intensity (MFI) of the cytokine producing cells was examined, this value was normalised to the cytokine negative cells within the same plot to minimise background fluorescence variation between samples.

### Statistical methods

Differences among the repeated measurements of FOXP3^+^ were evaluated using the Skillings-Mack test, which is the non-parametric equivalent of the one-way repeated measures ANOVA, and Wilcoxon signed-ranks tests for paired data with Bonferroni adjustment for multiple comparisons. P-values <0.05 were considered statistically significant. The correlation between the frequencies of FOXP3^+^ Treg and CEF-specific IFNγ/TNFα double producing T cells was calculated using a linear regression model (least squares fitting).

## Results and Discussion

### 1. Detection of multi-functional T cells by intracellular cytokine staining

**Figure 1:**
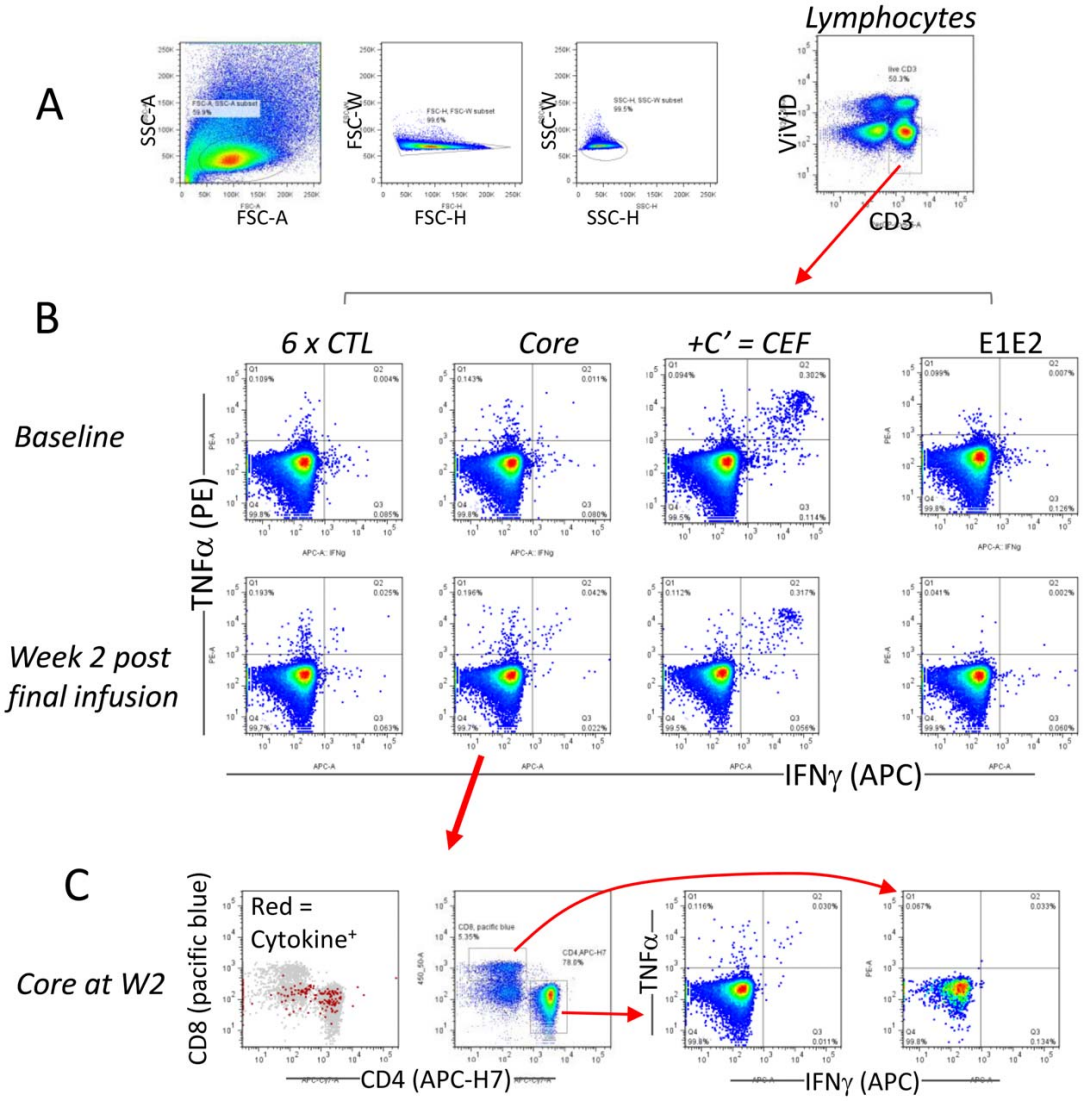
Gating strategy and example of ICS data. (**A**) Lymphocytes were gated based on FSC and SSC, followed by a CD3^+^ViViD^−^ viable T cell gate. (**B**) Example of data from PT#3, depicting typical positive (6×CTL, core antigen) and negative (E1E2 antigen) responses following DC infusion compared to baseline. (**C**) These plots are derived from the same data as the core antigen response at week 2 (W2), depicting the cellular source of the cytokines. The 1^st^ plot (left) shows cytokine positive cells (red) within viable CD3 T cells (grey), the 2^nd^ plot shows the position of the CD4^+^ and CD8^+^ T cell gates, and the 3^rd^ and 4^th^ plots depict the cytokine staining profiles of CD4^+^ T cells and CD8^+^ T cells respectively.

**Figure 2:**
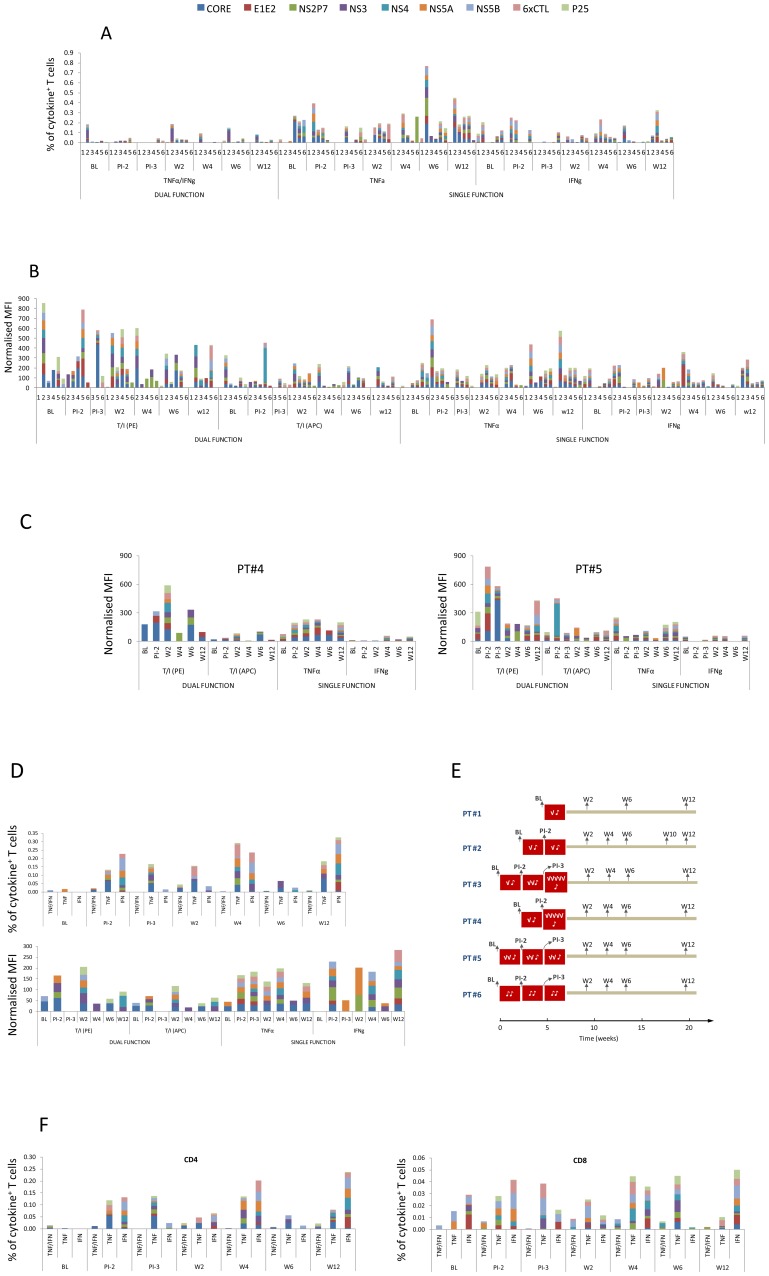
Cytokine positive responses - a complete dataset from all patients. (**A**) Cytokine producer frequency (%, gating on viable CD3^+^ T cells) at baseline and different time points thereafter. The numbers on the x-axis (1, 2, 3, 4, 5 and 6) are the patient ID. Abbreviations: BL = Base Line; PI-2 = Prior to 2^nd^ Infusion; PI-3 = Prior to 3^rd^ Infusion, W = Week post final infusion. (**B**) The normalised MFI, corresponding to the responses detected in (A). (**C**) T cell quality in PT#4 and PT#5, depicted by the normalised MFI of cytokine positive responses. (**D**) The frequency (%) and normalised MFI over time in PT#3. (**E**) The dose and timing of DC infusion each patient received and the timing of sample collection. (**F**) Contribution of CD4 and CD8 T cells (please note the differences in scale) to the cytokine positive responses in PT#3.

The Mo-DC dose each patient received and the viral load at various time points before and after the cell infusion were described previously [1]. In brief, our trial had 6 patients only, and as a dose escalation trial each patient received a different DC infusion dose and/or route (Table 1). We analysed PBMC from each patient in an independent experiment, in which all samples (various time points) from this patient were assayed in a single 96-well plate (to ensure that different PBMC samples from the same patient were compared directly) and the positive controls (to simply confirm the reactivity of the reagents) were assayed in a second plate. In addition, each assay also included a healthy donor PBMC sample as a negative control (to rule out false positives from the reagents). We gated on viable CD3^+^ T cells, with the help of ViViD dye (Invitrogen) [6] (Fig. 1A) and defined the cytokine positive cells with a generous clearance from the negative population (Fig. 1B). We then plotted the frequency of T cells according to their functional status, *viz*. TNFα or IFNγ single producer and TNFα/IFNγ double producer, as illustrated in Fig. 2A. To emphasize antigen-specific cytokine production, all data used to generate this figure were background subtracted using the no antigen control (the raw data without background subtraction are provided in Table S1).

**Table 1:** Summary of the dose escalation trial. 1 dose unit = 1×10^7^ DC.

*Patient ID*	*1^st^ infusion*	*2^nd^ infusion*	*3^rd^ infusion*
*# 1*	√ 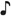		
*# 2*	√ 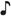	√ 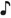	
*# 3*	√ 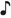	√√ 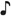	√√√√√ 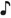
*# 4*	√ 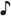	√√√√√ 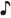	
*# 5*	√√ 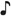	√√ 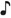	√√ 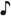
*# 6*	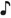 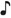	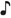 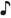	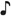 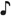

*(√ = 1 dose unit infused via i.v route and 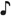 = 1 dose unit infused via i.d route).*

*(Adapted from Gowans et al. (2010) J Hepatol; 53:599).*

We found that single and/or double positive T cells were detected in every patient, although the timing and the exact HCV protein specificity varied. The quality of the responses was poor, and mostly comprised cells which secreted a single cytokine. This is in sharp contrast to the *in vitro* positive control CEF, which induced strong IFNγ/TNFα double positive responses, sometimes in the absence of single positive responses (Figure S1). Within the single cytokine category, TNFα producers showed a higher frequency compared to IFNγ producers, and in some patients (such as PT#2) these cells showed an increasing trend post vaccination (Fig. 2A). Although few in number, on a per cell basis the dual positive cells produced higher levels of cytokines, reflected by an overall higher MFI, in particular in the TNFα PE channel (Fig. 2B). All MFI used in this study were normalised as outlined in the method section. These data are consistent with the notion that multi-functional T cells are superior to single functional T cells [3], [9]. These data also highlight the fact that induction of multi-functional T cells is highly desirable for HCV vaccine design. Unfortunately, apart from some isolated examples, for instance the increase in MFI post vaccination in PT#4 and PT#5 (plotted individually in Fig. 2C), we did not observe an overt improvement in T cell quality by the Mo-DC therapy.

Upon further inspection we noted an increase in T cell responses post vaccination in PT#3 (Fig. 2D). There was an increase over time post DC infusion not only in the frequency of cytokine producing cells (upper panel), but also in the level of cytokine each cell produce (lower graph). Remarkably, this improvement occurred in all three cytokine categories, *viz*. the IFNγ single, TNFα single and TNFα/IFNγ dual function, which was not observed in the other patients. Interestingly, patient #3 received the highest DC dose (Fig. 2E). We also found that this increase was mainly contributed by CD4^+^ T cell responses (Fig. 2F, note the different scales for CD4 and CD8 T cells), supporting the aim of the trial to enhance CD4^+^ T cell responses. While a similar trend was not apparent in every patient, in PT#2 and PT#6 the increase in TNFα single positive cells was quite remarkable (Fig. 2A). Although there may be some degree of non-specific enhancement by Mo-DC infusion on cytokine production, compared to the HCV-specific responses (Table S2), CEF-specific responses were rather stable after DC vaccination, with the index to baseline close to 1 (Table S3). Taken together, these data suggest that a boost in HCV-specific T cell responses was likely achieved by Mo-DC therapy in at least some patients, although the overall pattern was consistent with our previous report [1] in that the responses were sporadic, inconsistent among patients and showed no apparent correlation with time or immunization dose/route. We recognise that this Phase I trial had a small sample size and consequently it was not feasible to apply a stringent cut-off for positive responses. This has left potential biases introduced via background subtraction unchecked. It would be valuable for these results to be confirmed using a larger patient cohort and thus the data should be considered with caution.

Both CD4^+^ and CD8^+^ T cells contributed to the positive responses, however, since CD4^+^ cells were always more abundant than CD8^+^ T cells, the net number of cytokine-producing CD4^+^ T cells was usually greater than that of CD8^+^ T cells (Figure S2A). The cellular source of cytokines also included CD3^+^ but not typical CD4^+^ or CD8^+^ T cells (Fig. 1C and Figure S2B), and the exact nature of these cells was unclear. We noticed discrepancies between our ICS data in this study and our previous ELISPOT data, and this issue is addressed further in the supporting information (please see Data S1), where we demonstrate, as well as discuss, that while not fundamentally conflicting, ICS and ELISPOT were not quantitatively comparable. Consistent with the previous study we found that positive cytokine responses were detected in response to viral antigens that were not included in the cellular vaccine (for example the response to NS2P7 in PT#6 and to NS5A in PT#3), suggesting that Mo-DC therapy may induce broader responses via epitope spreading [1] or other undefined mechanisms. RT-PCR amplification of the circulating virus RNA followed by sequencing confirmed that the peptides contained in the vaccine matched and thus discrepancies between vaccine and virus did not account for the poor immune responses [1]. We could not detect any IL2^+^ T cells, other than after PMA/ionomycin stimulation in positive control cultures (not shown), probably because the HCV peptide stimuli were insufficient.

### 2. Mo-DC therapy in HCV patients may influence FOXP3^+^ Treg frequency

**Figure 3:**
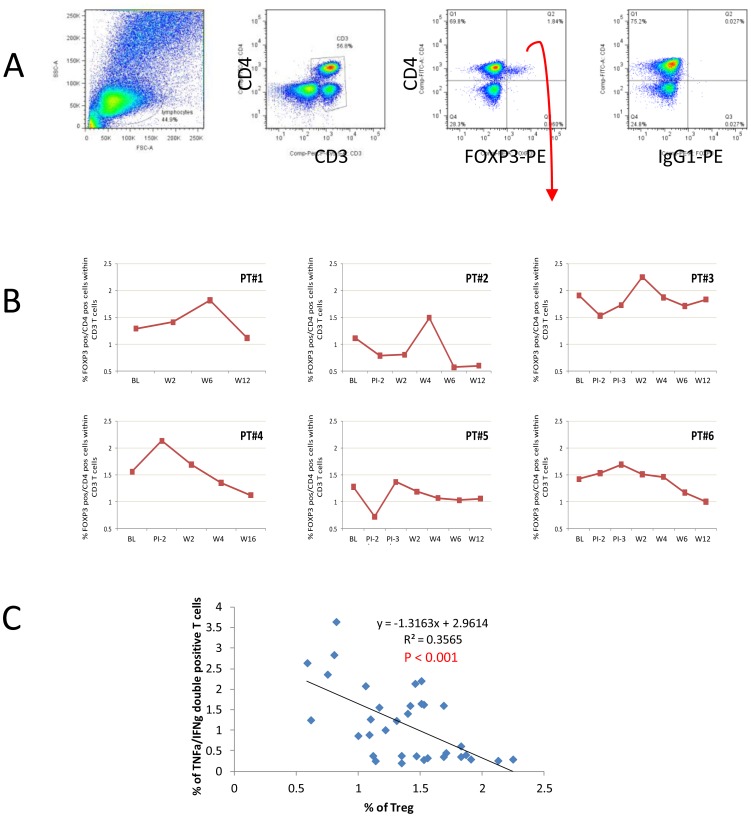
T cells expressing FOXP3. (**A**) Example of plots showing gate settings. (**B**) The frequency of FOXP3^+^ T cells at different time points, gating on CD3^+^ T cells. (**C**) Inverse correlation between the frequencies of FOXP3^+^ Treg and CEF-specific TNFα/IFNγ dual functional T cells.

Recent work by us [8], [10] and others (reviewed in [11]) suggested that Treg may be implicated in HCV persistence and HCV-specific Treg have been detected using tetramers [5], [7], [11], [12]. We therefore investigated the presence of Treg in patients in this trial. In each patient the frequency of FOXP3^+^ Treg fluctuated at early time points following DC infusions, but decreased to below baseline by week 12 (Fig. 3, Table 2 and Table 3). If we compare values before any DC infusion (baseline) and after the final infusion, there was a statistically significant difference in the frequency of FOXP3^+^ Treg (P = 0.013, Table 3). In particular, the week 12 median frequencies tended to be lower than baseline values although not significant when adjusted for multiple comparisons (1.385 to 1.09, P = 0.172, Table 3, while before adjustment P = 0.043, data not shown). Due to the small sample size, the power to detect any significant differences is limited, but collectively, the frequency of Treg at week 12 displayed a trend of reduction compared to baseline, offering exciting hints for future follow-up studies.

**Table 2:** The frequency (% within CD3^+^ T cells) of FOXP3^+^ Treg of the patients at various time points.

	*BL*	*PI-2*	*PI-3*	*W2*	*W4*	*W6*	*W12*
*PT#1*	1.35			1.47		1.83	1.14
*PT#2*	1.13	0.806		0.823	1.51	0.589	0.618
*PT#3*	1.91	1.53	1.73	2.25	1.87	1.71	1.83
*PT#4*	1.56	2.13		1.69	1.35	1.12	*N/D*
*PT#5*	1.31	0.753	1.4	1.22	1.1	1.06	1.09
*PT#6*	1.42	1.53	1.69	1.51	1.46	1.17	1
*Mean*	1.447	1.35	1.61	1.494	1.458	1.247	1.136
*Median*	1.385	1.53	1.69	1.49	1.46	1.145	1.09
*SD*	0.27	0.58	0.18	0.48	0.28	0.46	0.44

**Table 3:** Skillings-Mack and Wilcoxon signed-rank test (with Bonferroni adjustment) for FOXP3 data (see Table 2).

*Comparison*	*Test*	*P-value*
*Baseline, Week 2, 4, 6, 12 only*	Skillings-Mack test	0.013*
*BL vs wk12*	Wilcoxon signed-ranks test	0.172
*BL vs wk6*	Wilcoxon signed-ranks test	0.992
*BL vs wk4*	Wilcoxon signed-ranks test	1.000
*BL vs wk2*	Wilcoxon signed-ranks test	1.000

It has been reported that Mo-DC infusions induced FOXP3^high^ regulatory T cells, and depletion of CD25^+^ cells enhanced the efficacy of Mo-DC-based immunotherapy in cancer patients [12], [13]. Our data suggest a different outcome, which can likely be explained by a combination of differences in pathogenesis and the design of the Mo-DC therapy. It is possible that this reduction is temporary, within the time frame of the trial. We speculate that the decrease in FOXP3 Treg is related to the increase in TNFα single positive cells that showed a trend towards up-regulation after the Mo-DC therapy. It was recently discovered that TNFα, which signals through TNFRII expressed by Treg [14], [15], is an important regulator of Treg function in mice and humans, and can variously promote or inhibit Treg activity in a dose-dependent fashion [16], [17]. It is therefore an intriguing possibility that vaccine-induced HCV-specific and/or HCV-non-specific TNFα production by effector T cells may temporarily reduce Treg activity in these patients. We cannot exclude the possibility that some FOXP3^+^ cells, especially during the early time points following the initial infusion, may be *in vivo* activated conventional T cells, as although FOXP3 is the best marker available for Treg it is less specific in humans compared with mice [11], [18]. However, the frequency of FOXP3^+^ Treg was inversely correlated (P<0.001) with that of CEF-specific responses (Fig. 3C), suggesting that the FOXP3^+^ cells are principally Treg.

Collectively, these data suggested that our Mo-DC therapy was capable of inducing multi-functional T cells, although there was no consistent response related to either the timing, post DC infusion, or the specific HCV antigen. The presence of Treg may however complicate the *in vivo* immune response induced by the treatment. Thus the phase I Mo-DC immunotherapy trial showed multiple outcomes, but overall was unable to generate sustained cell-mediated immune responses to break tolerance to HCV. Alternative strategies to examine longer term therapy, different antigens, vaccination routes, Treg manipulation or increased dosage may need to be considered to achieve therapeutic benefit.

## Supporting Information

Figure S1 **The frequency of cytokine producing T cells in the** ***in vitro*** **positive control CEF peptide pool stimulated culture.** All data were background subtracted using the no antigen control. Analysis gated on viable CD3^+^ T cells.
(PDF)

Figure S2 **Cytokine production by CD4 and/or CD8 T cells.** (**A**) Heat map depicting the frequency of cytokine producing CD4^+^ and CD8^+^ T cells within viable (ViViD^−^) CD3^+^ T cells. Data are background subtracted, and only positive values are retained and coloured accordingly. (**B**) An example of positive response (NS2P7 response in PT#6), depicting the cellular source of the cytokines. The red dots represent cytokine^+^ T cells and the grey dots are viable CD3^+^ T cell.
(PDF)

Table S1 **Frequency (%) of cytokine producing T cells within total viable CD3^+^ T cells, before background subtraction.**
(XLSX)

Table S2 **Background subtracted data for HCV antigens.** A) Percentage of cytokine producing T cells within total viable CD3^+^ T cells, background subtracted, and retaining positive values only. B) Index to BL.
(XLSX)

Table S3 **Background subtracted data for** ***in vitro*** **positive control CEF.** A) Percentage of cytokine producing T cells within total viable CD3^+^ T cells, background subtracted, and retaining positive values only. B) Index to BL for TNFα/IFNg dual functional T cells.
(XLSX)

Data S1 **Discrepancies between ELISpot and ICS data.**
(PDF)
