# Supplementary material for: Induction of Multi-Functional T Cells in a Phase I Clinical Trial of Dendritic Cell Immunotherapy in Hepatitis C Virus Infected Individuals
Source: PLoS One. 2012 Aug 14;7(8):e39368. doi: 10.1371/journal.pone.0039368 (PMC3419178; doi:10.1371/journal.pone.0039368)

Figure S1. The frequency of cytokine producing T cells in the *in vitro* positive control CEF peptide pool stimulated culture, background subtracted using the no antigen control. Analysis gated on viable $CD3^{+}$ T cells.

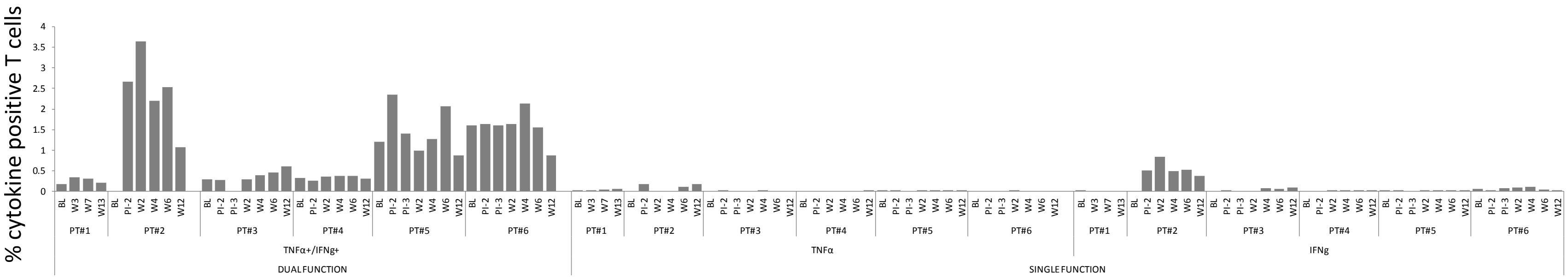

Supplement: Figure S1 — The frequency of cytokine producing T cells in the in vitro positive control CEF peptide pool stimulated culture. All data were background subtracted using the no antigen control. Analysis gated on viable CD3+ T cells. (PDF) [file pone.0039368.s001.pdf]
