# Supplementary material for: Induction of Multi-Functional T Cells in a Phase I Clinical Trial of Dendritic Cell Immunotherapy in Hepatitis C Virus Infected Individuals
Source: PLoS One. 2012 Aug 14;7(8):e39368. doi: 10.1371/journal.pone.0039368 (PMC3419178; doi:10.1371/journal.pone.0039368)
Supplement: Figure S2 — Cytokine production by CD4 and/or CD8 T cells. (A) Heat map depicting the frequency of cytokine producing CD4+ and CD8+ T cells within viable (ViViD−) CD3+ T cells. Data are background subtracted, and only positive values are retained and coloured accordingly. (B) An example of positive response (NS2P7 response in PT#6), depicting the cellular source of the cytokines. The red dots represent cytokine+ T cells and the grey dots are viable CD3+ T cell. (PDF) [file pone.0039368.s002.pdf]

Figure S2. Cytokine production by CD4 and/or CD8 T cells (A) Heat map depicting frequency of cytokine producing $CD4^+$ and $CD8^+$ T cells within viable $CD3^+$ T cells. All data are background subtracted, and only positive values are retained. . **(B)** An example of positive response (NS2P7 response in PT#6), depicting the cellular source of the cytokines. The red dots represent cytokine$^+$ T cells and the grey dots are ViViD$^-$ $CD3^+$ T cell.

A

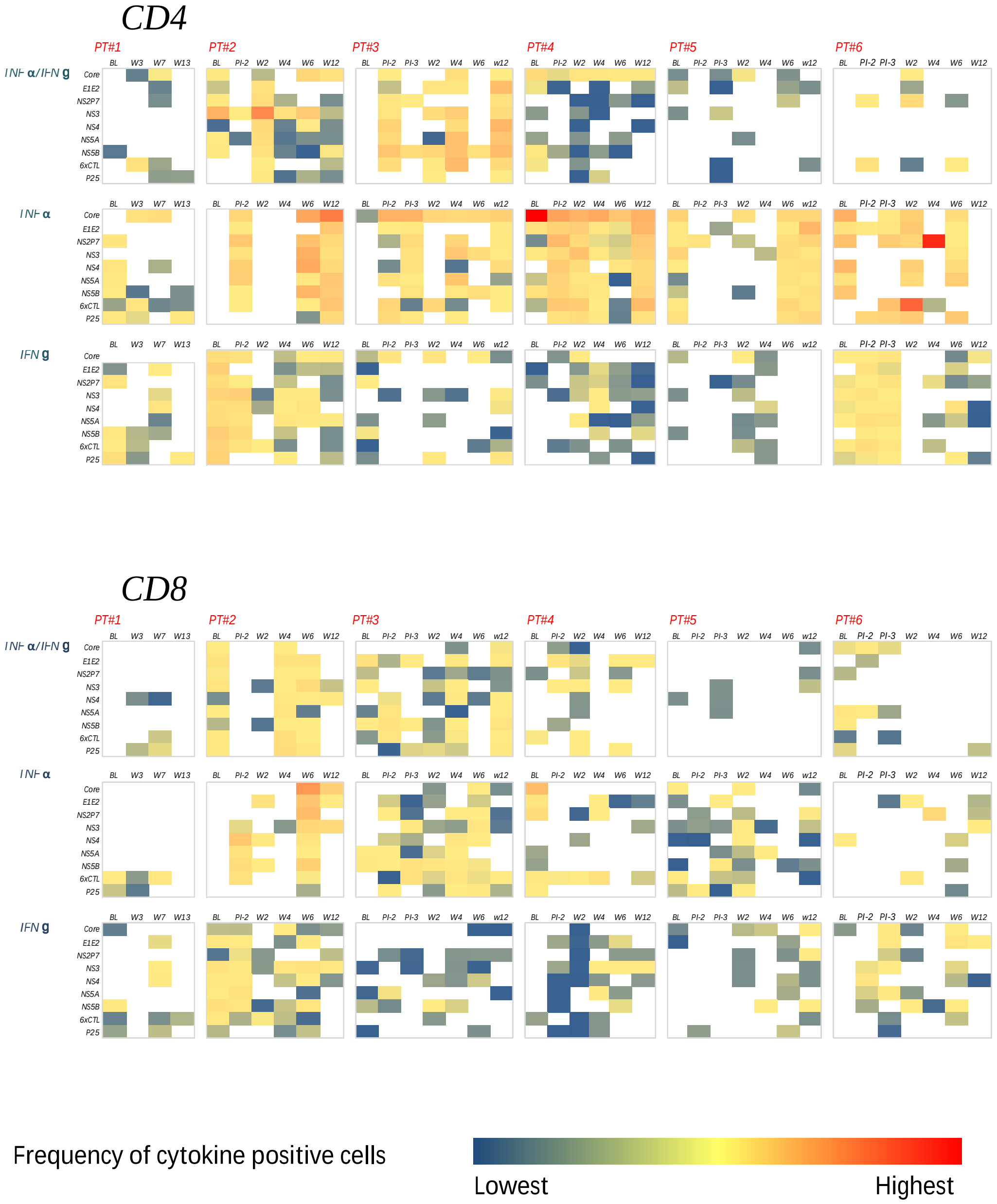

B

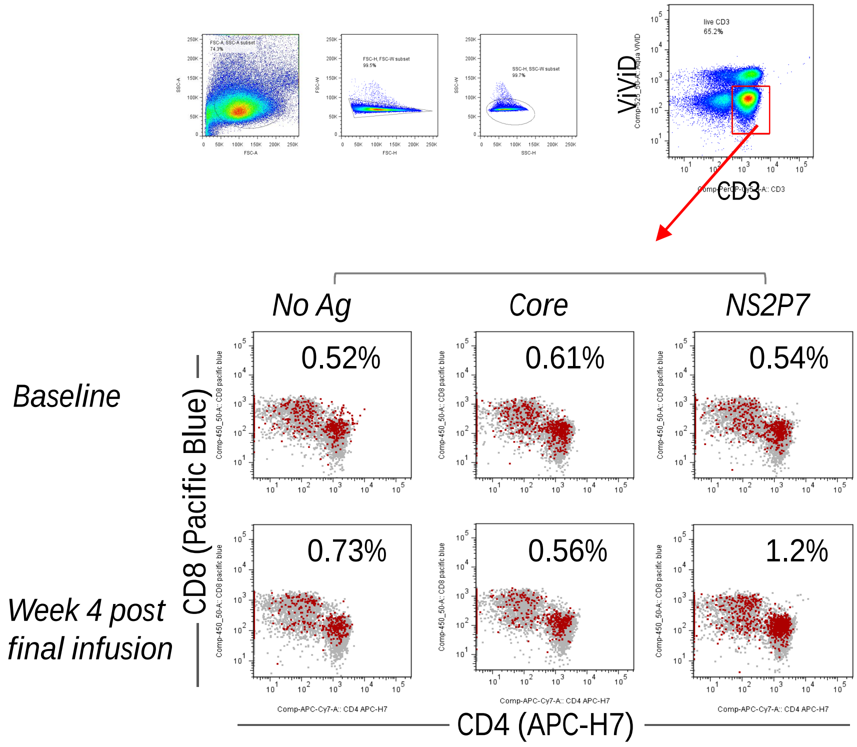

live CD3
65.2%
ViViD
CD3
No Ag
Core
NS2P7
Baseline
Week 4 post
final infusion
0.52%
0.61%
0.54%
0.73%
0.56%
1.2%
CD8 (Pacific Blue)
CD4 (APC-H7)
Comp-APC-Cy7-A:: CD4 APC-H7
Comp-450_50-A:: CD8 pacific blue
