# Supplementary material for: Induction of Multi-Functional T Cells in a Phase I Clinical Trial of Dendritic Cell Immunotherapy in Hepatitis C Virus Infected Individuals
Source: PLoS One. 2012 Aug 14;7(8):e39368. doi: 10.1371/journal.pone.0039368 (PMC3419178; doi:10.1371/journal.pone.0039368)
Supplement: Data S1 — Discrepancies between ELISpot and ICS data. (PDF) [file pone.0039368.s006.pdf]

**Supplementary data: Discrepancies between ELISpot and ICS data**

Since ELISPOT and ICS both aim to quantify cytokine producing cells, one would expect that they should have comparable results. We found however this was not the case.

In this section, we first compare the data, and then discuss why there should be inconsistencies, yet collectively have statistical correlation.

*How to compare?*

*1)* In ELISPOT we used whole PBMC, at 100,000 per well. In ICS, data could not be meaningfully analysed without previous gating, and the closest gate that we believe to resemble whole PBMC is live lymphocytes (in this figure below, the red oval gate in C).

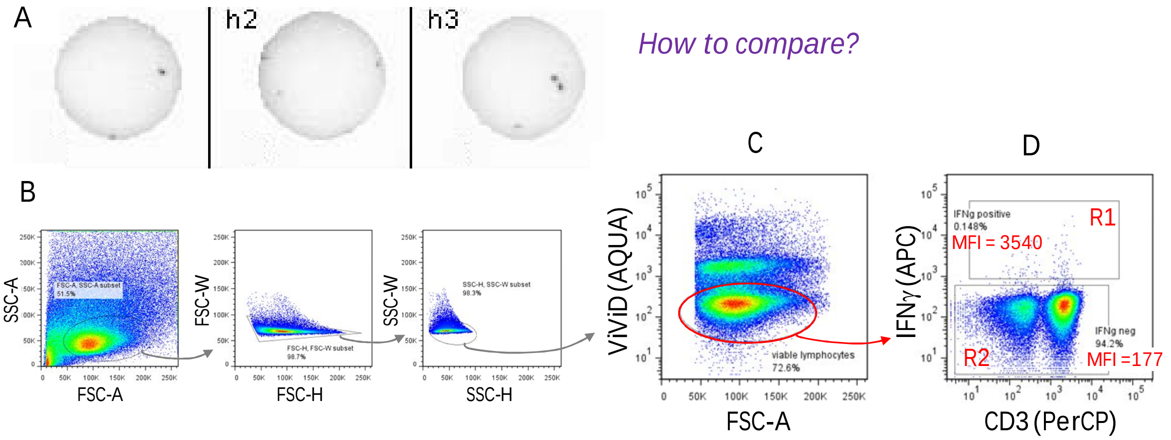


$$TFI_{NORM} = (\% \text{ of } IFN\gamma^{+}) \times (MFI \text{ of } IFN\gamma^{+}) \, / \, (MFI \text{ of } IFN\gamma^{-})$$

*2)* In ELISPOT (panel A in the above figure), a single value represents the number of cytokine producing cells (the number of spots) and the amount of cytokine produced (represented by the size of the spot). In ICS (see plot D above), this information is in two separate values: the percentage of positive cells and the mean fluorescent intensity (MFI) of the cells. To compare ICS to ELISPOT we need to first integrate the percentage and MFI into one value. This may be best achieved by the normalised Total Florescent Intensity ($TFI_{NORM}$, please see reference [8, 10], where $TFI_{NORM}$ was used in HCV studies), which is the percentage of cytokine positive cells (R1 in plot D) multiplied by MFI of these cells and then normalised to the MFI of the negative population (R2). In the example above, $TFI_{NORM}$ = 0.148 × 3540 / 177 = 2.96.

*3)* In this comparison, for ICS we used fluorescent intensity data for IFNγ only, as the ELISPOT only measured this cytokine.

*4)* In ELISPOT, all the data are background subtracted without additional cut off. We did the same for $TFI_{NORM}$, as illustrated below.

*$TFI_{NORM}$ of PT#6, background subtracted, retaining positive values only*

| | BL | PI-2 | PI-3 | W2 | W4 | W6 | W12 |
|---|---|---|---|---|---|---|---|
| CORE | | | | 0.38 | | | |
| E1E2 | | 0.21 | 0.25 | 0.52 | | | |
| NS2P7 | | 0.38 | | 0.34 | 0.03 | | |
| NS3 | | | | 0.21 | 0.32 | | |
| NS4 | | | | 1.62 | | | |
| NS5A | | | | 0.30 | | | |
| NS5B | 0.21 | | | | | | |
| 6xCTL | | 1.91 | 0.35 | 1.01 | 0.12 | | |
| P25 | | | 0.09 | | | | |
| No Ag | | | | | | | |
| CEF | 169.03 | 154.26 | 148.03 | 164.93 | 191.01 | 154.19 | 85.80 |

*5)* The Core, NS3, NS5A and NS5B peptide pools were used in an identical fashion in ICS and ELISPOT, but we combined some of the peptide pools for ICS that were previously used individually in ELISPOT, for example, NS2 and P7 were used individually in ELISPOT but combined (named NS2P7 now) in ICS. Subsequently, the ELISPOT "spots per million PBMC" values from NS2 and P7 were summed to enable the comparison. This principle also applies to E1E2, NS4 and 6×CTL peptides.

*Results*

For each patient, $TFI_{NORM}$ for ICS and spots per million PBMC for ELISPOT (values are summed when required, as explained above) are presented in the heat map below, all the values are background subtracted (and only positive values are retained, and colour coded). **The two sets of data showed little consistency.**

*Comparing ELISPOT and ICS data.*

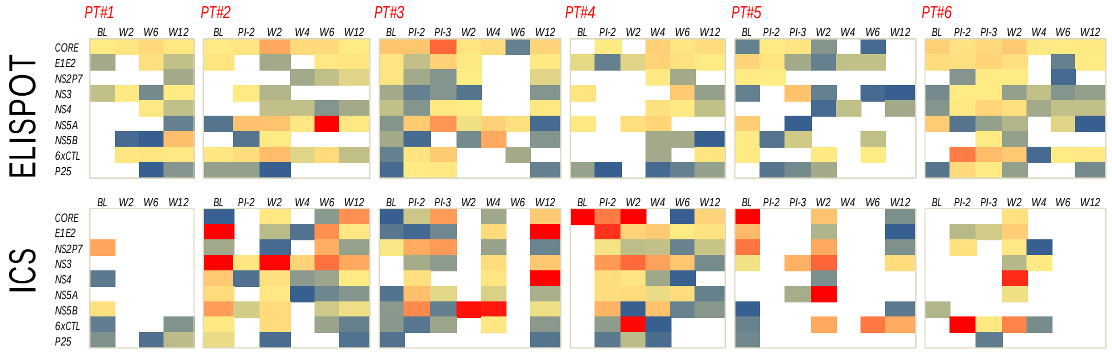


*Colour code for the values:*

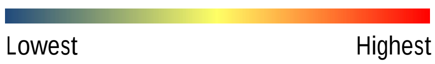

*Discussions*

Why? Theoretically, multiple factors contribute to this discrepancy, for example:

***a)*** ELISPOT measures the amount of cytokine secreted by a cell, whereas ICS measures the cytokine blocked within the cell. The two quantities can be quite different;

***b)*** ELISPOT and ICS process the information differently. Although the size of the spots and fluorescent intensity should both correlate with the amount of cytokine produced, the quantitative relationship between the two values is unknown, but since ELISPOT is detectable by the camera and ICS by laser, it is expected that the detection threshold for a positive cell can be very different. Thus the distribution co-efficiency of the size or intensity values affects the two data differently;

***c)*** The assays are quite different in that the stimulation time for ELISPOT is 20 hours while for ICS is 6 hours (Golgi blockers are toxic to cells if used for too long), thus the kinetics of cytokine production may influence the data.

In a general sense? When data from all patients were combined and the total number of responses detected at each time point were counted (regardless of the magnitude, please see reference [7], where ICS responses were counted in this manner), we discovered a significant positive correlation between ELISPOT and ICS (P = 0.018, Wilcoxon signed-ranks test). Thus in a broad and collective sense, **ICS does not fundamentally conflict with ELISPOT.**

*Total numbers of cytokine positive responses detected at each time point, data from all patients are summed.*

| | *BL* | *PI-2* | *PI-3* | *W2* | *W4* | *W6* | *W12* |
|---|---|---|---|---|---|---|---|
| *by ELISPOT* | 34 | 31 | 23 | 36 | 29 | 35 | 39 |
| *by ICS* | 30 | 23 | 12 | 33 | 24 | 17 | 32 |

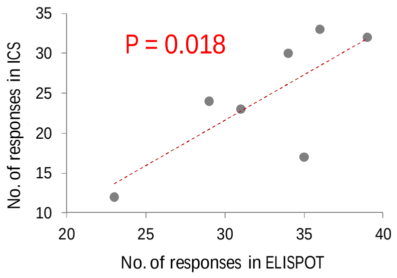


Thus, while not fundamentally conflicting, the ELISPOT and ICS data could not be quantitatively compared. We believe that this situation has a theoretical basis and could not be resolved by repeating the experiments side by side.
